# Assessment of genotypes, endosymbionts and clinical characteristics of *Acanthamoeba* recovered from ocular infection

**DOI:** 10.1186/s12879-022-07741-4

**Published:** 2022-09-29

**Authors:** Binod Rayamajhee, Savitri Sharma, Mark Willcox, Fiona L. Henriquez, Raksheeth Nathan Rajagopal, Gauri Shankar Shrestha, Dinesh Subedi, Bhupesh Bagga, Nicole Carnt

**Affiliations:** 1 School of Optometry and Vision Science, Faculty of Medicine and Health, UNSW, Sydney, Australia; 2 Jhaveri Microbiology Centre, Prof. Brien Holden Eye Research Centre, Hyderabad Eye Research Foundation, L. V. Prasad Eye Institute (LVPEI), Kallam Anji Reddy Campus, Hyderabad, India; 3 Institute of Biomedical and Environmental Health Research, School of Health and Life Sciences, University of the West of Scotland (UWS), Paisley, PA1 2BE Scotland, UK; 4 The Cornea Institute, L V Prasad Eye Institute, Banjara Hills, Hyderabad, India; 5 School of Biological Sciences, Monash University, Clayton, VIC 3800 Australia

**Keywords:** *Acanthamoeba*, Keratitis, Genotyping, Endosymbionts

## Abstract

**Introduction:**

*Acanthamoeba* is an emerging pathogen, infamous for its resilience against antiprotozoal compounds, disinfectants and harsh environments. It is known to cause keratitis, a sight-threatening, painful and difficult to treat corneal infection which is often reported among contact lens wearers and patients with ocular trauma. *Acanthamoeba* comprises over 24 species and currently 23 genotypes (T1-T23) have been identified.

**Aims:**

This retrospective study was designed to examine the *Acanthamoeba* species and genotypes recovered from patients with *Acanthamoeba* keratitis (AK), determine the presence of endosymbionts in ocular isolates of *Acanthamoeba* and review the clinical presentations.

**Methodology:**

Thirteen culture-confirmed AK patients treated in a tertiary eye care facility in Hyderabad, India from February to October 2020 were included in this study. The clinical manifestations, medications and visual outcomes of all patients were obtained from medical records. The *Acanthamoeba* isolates were identified by sequencing the ribosomal nuclear subunit (*rns*) gene. *Acanthamoeba* isolates were assessed for the presence of bacterial or fungal endosymbionts using molecular assays, PCR and fluorescence in situ hybridization (FISH).

**Results:**

The mean age of the patients was 33 years (SD ± 17.4; 95% CI 22.5 to 43.5 years). Six (46.2%) cases had AK associated risk factors; four patients had ocular trauma and two were contact lens wearers. *A. culbertsoni* (6/13, 46.2%) was the most common species, followed by *A. polyphaga* and *A. triangularis*. Most of the isolates (12/13) belonged to genotype T4 and one was a T12; three sub-clusters T4A, T4B, and T4F were identified within the T4 genotype. There was no significant association between *Acanthamoeba* types and clinical outcomes. Eight (61.5%) isolates harboured intracellular bacteria and one contained *Malassezia restricta*. The presence of intracellular microbes was associated with a higher proportion of stromal infiltrates (88.9%, 8/9), epithelial defect (55.6%, 5/9) and hypopyon (55.6%, 5/9) compared to 50% (2/4), 25% (1/4) and 25% (1/4) AK cases without intracellular microbes, respectively.

**Conclusions:**

Genotype T4 was the predominant isolate in southern India. This is the second report of T12 genotype identified from AK patient in India, which is rarely reported worldwide. The majority of the *Acanthamoeba* clinical isolates in this study harboured intracellular microbes, which may impact clinical characteristics of AK.

**Supplementary Information:**

The online version contains supplementary material available at 10.1186/s12879-022-07741-4.

## Introduction

*Acanthamoeba* keratitis (AK) is a rare eye disease accounting for 2% of global corneal infections [1]. However, perhaps due to increase in contact lens use and increasing prevalence of *Acanthamoeba* species in different water resources including man-made swimming pools and even treated domestic water supplies [2], AK cases are increasing globally. Contact lens wear is increasing worldwide in part due to the development of contact lenses that can control the progression of myopia in children, and this may put them at risk of developing AK infections that can lead to blindness [3]. The link between AK and contact lens wear is firmly established with contact lens wear being associated with nearly 90% of the reported infections [4]. Reported outbreaks have been linked to ineffective contact lens disinfecting solutions [5, 6]. The lifecycle of *Acanthamoeba* includes an infective trophozoite and the dormant cyst stage; the latter being able to remain viable for a number of years [7].

AK is difficult to diagnose and there are very limited effective treatment regimens [8, 9]. *Acanthamoeba* cysts are resilient to disinfectants, anti-protozoal drugs and nutrient depletion, posing a formidable challenge for patient care [10]. Furthermore, many of the common drugs to treat eye infection are not effective for *Acanthamoeba*. As the diagnosis and management of AK patients is difficult, this can lead to lengthy medication and successful treatment becomes extremely difficult leading to substantial vision loss [11]. Surgical interventions are needed in 30% of the AK patients to control the disease and in rare cases infected eye is removed [12]. Furthermore *Acanthamoeba* cysts are difficult to eradicate after infection has been established, and this can result in recurrence of infection [13]. Correct diagnosis is essential for successful therapy, but as the clinical signs and symptoms of AK vary and some are similar to other ocular infections such as herpes simplex virus (HSV) keratitis, diagnosis can be challenging. Pain relief, anti-inflammatories and general antimicrobial compounds are likely to be prescribed while diagnosis is established. Prior use of topical corticosteroid before diagnosis of *Acanthamoeba* corneal infection is associated with poorer visual outcome [14]. AK with suspected resistance to multipurpose contact lens solutions and established anti-amoebal remedies necessitate sensitive diagnostic modalities with novel therapeutic approaches [15].

Based on cell morphology, which can be influenced by culture conditions and source of isolation, *Acanthamoeba* spp. are classified into 3 groups, with keratitis causing strains most commonly belonging to group II [7]. At least 23 genotypes (T1-T23) have been identified based on the *Acanthamoeba* 18S rRNA gene sequence and the common keratitis causing species such as *A. castellani* and *A. polyphaga* are of clade T4 [16]. Genotypes T2, T3, T5, T6, T10, T11, T12, T13, T15, and T16 have also been recovered from AK patients, although more rarely [17–19]. Inter-strain variations in the *Acanthamoeba* 18S rRNA subunit (*rns*) gene sequence can be used to identify sub-generic genotypes [20].

*Acanthamoeba* is a heterotrophic protist that can be host to bacteria, fungi and giant viruses [21, 22]. Coinfections of *Acanthamoeba* with other pathogens such as *Fusarium* spp. or *Pseudomonas aeruginosa* have been observed in keratitis patients [23]. These coinfections could be due to the intracellular carriage of these pathogens within the infecting *Acanthamoeba.* Furthermore, *Acanthamoeba* can act as a training ground for bacterial pathogens to invade higher eukaryotic cells [24]. What is not known is how these intracellular microorganisms affect the treatment and outcomes of AK disease but studies have reported that presence of intracellular microbes enhances the pathogenicity of *Acanthamoeba* isolates [25, 26]. In the present study, 13 *Acanthamoeba* isolates recovered from AK patients in southern India were examined to gauge the epidemiological distribution of *Acanthamoeba rns* genotypes and sub-genotypes and their clinical manifestations. The presence of intracellular bacteria and fungi in *Acanthamoeba* isolates was assessed and determinations made of whether these were associated with clinical outcomes of AK patients.

## Methods

A hospital-based retrospective case series study was conducted from hospital records and *Acanthamoeba* strains isolated at LV Prasad Eye Institute (LVPEI), Hyderabad, India from February to October 2020. This study protocol was reviewed and approved by the institutional review board of LVPEI, Hyderabad (LEC-BHR-R-09–21-758). Thirteen *Acanthamoeba* isolates recovered from AK patients were included in this study.

### Culture and microscopic assessment of *Acanthamoeba*

During the study period, as per institutional protocol, all patients presenting with clinical features of microbial keratitis underwent a comprehensive slit-lamp examination for investigation of the corneal epithelial defect, size of infiltrate (if present), size of hypopyon, involvement of sclera, absence/presence of foreign particles and documentation of the ocular adnexal status [27]. The microbiological investigation of the corneal scrapings included smear preparation for microscopy using Gram stain and potassium hydroxide with calcofluor white (KOH + CFW) mount, and inoculation into appropriate media [5% sheep blood agar (BA), chocolate agar (CA), Sabouraud dextrose agar (SDA), potato dextrose agar (PDA), non-nutrient agar with *Escherichia coli* (NNA), thioglycolate broth, and brain heart infusion broth (BHI)]. Smear and inoculation of all media were done (as is standard) by the attending clinician at the slit lamp. Dehydrated culture media were supplied by HiMedia, Mumbai, India and freshly prepared in-house for use. Bandage contact lenses (2/13) were aseptically cut into small pieces and inoculated into BA, CA, SDA, NNA and BHI in the microbiology laboratory. All media were incubated aerobically at 37 °C except for SDA and PDA at 27 °C and chocolate agar which was incubated in 5% CO2 at 37 °C. The media were observed for 14 days for any growth. *Acanthamoeba* isolates from corneal scraping of 13 patients were confirmed by observation of active trophozoites and double-walled polygonal cysts; confirmed isolates were preserved on NNA and transported to School of Optometry and Vision Science, Faculty of Medicine and Health, UNSW, Sydney, Australia. The thermotolerance of isolates was evaluated by growing isolates at 28 °C, 37 °C, 40 °C and 42 °C. Following initial isolation, the *Acanthamoeba* strains were grown axenically in peptone yeast glucose (PYG) medium [28]. Each culture plate was monitored to ensure no extracellular microbes or contamination was present. To prevent possible contamination the culture medium was replaced with freshly prepared PYG every two days till trophozoites were harvested. In addition, a separate sterile incubator set at 32 °C was used for this study.

### Genomic DNA (gDNA) extraction, PCR and 18S rRNA sequencing of *Acanthamoeba*

*Acanthamoeba* strains were further identified by PCR assay. Amoebal cells grown on NNA were collected into 500µL of 1X PBS (1.4 mM NaCl, 2.7 mM KCl, 10 mM Na_2_HPO_4_ and 1.8 mM KH_2_PO_4_, pH 6.9) using a sterile scraper (Sigma-Aldrich, St. Louis, Missouri, USA). Genomic DNA (gDNA) was extracted using 10% v/v Chelex lysis solution (Bio-Rad, CA, USA,) in 0.1% v/v Triton X-100 (Sigma-Aldrich) and Tris buffer, pH 8.0 (Thermo Fisher Scientific, Bedford, USA), as previously explained [29]. The quantity of extracted dsDNA was measured using Nano Drop UV–Vis spectrophotometer (Thermo Fisher Scientific) and gDNA vials were stored at -20 °C until further use.

The *rns* gene region of 18S rRNA was amplified in a PCR reaction using *Acanthamoeba* genus specific primers, JDPFw (5’-GGCCCAGATCGTTTACCGTGAA-3’) and JDPRv (5’-TCTCACAAGCTGCTAGGGGAGTCA-3’), which encode the highly variable DF3 region and give amplicons of ~ 450 bp [30]. Each PCR assay was performed in 12.5µL of DreamTaq Master Mix (DNA Polymerase, 2X DreamTaq buffer, dATP, dCTP, dGTP and dTTP: 0.4 mM each, and 4 mM MgCl_2_; Thermo Fisher Scientific), 1µL of each primer (10 µM), 6.5µL of molecular grade water and 4µL of DNA template. PCR amplification was carried out in a 96-well T100 thermal cycler (Bio-Rad, California, USA) using the following thermal cycling conditions: 95 °C for 5 min for initial denaturation, followed by 35 cycles of amplification (94 °C for 30 s, 56 °C for 30 s and 72 °C for 45 s) and a final extension at 72 °C for 10 min [31]. PCR amplifications were observed by electrophoresis of 4μL PCR product aliquots in 1% agarose gel and amplicon bands were examined using Gel Doc XR + with image lab software (Bio-Rad). PCR positive amplicons were sent to the Ramaciotti Centre for Genomics (UNSW, Sydney, Australia) for Sanger sequencing. PCR products were purified using an EXOSAP-IT kit (Thermo Fisher Scientific) and sequencing was performed with forward primer JDPFw (5’-GGCCCAGATCGTTTACCGTGAA-3’) using BigDye Terminator (V3.1) reaction mix in 3730 DNA analyser (Applied Biosystems, Massachusetts, USA).

### Genotype and species identification of *Acanthamoeba* isolates

Low quality nucleotide sequences were manually checked and trimmed with Chromas (2.6.6) software (Technelysium Pty Ltd, Brisbane, Australia) [32]. The trimmed sequence reads were blast in the NCBI nucleotide sequences database (BLASTn) to identify the *Acanthamoeba* genotypes and species. Additionally, isolated *Acanthamoeba* strains were morphologically characterized to the species based on the classification scheme of Pussard and Pons [33]. All the confirmed and validated sequence reads were submitted to GenBank sequence data repository. Publicly available nucleotide sequences of *Acanthamoeba* genotypes were retrieved from the NCBI as reference strains for phylogenetic analysis (Additional file 1: Table S1). Sequences were aligned using ClustalW algorithm and a phylogenetic tree was constructed using the maximum likelihood method and Bayesian approach with Kimura-2 parameters by 1,000 bootstraps in MEGA-X [34] and phylogenetic tree was visualised using the interactive tree of life (iTOLv6) [35].

### Genomic nucleic acid extraction targeting endosymbionts of *Acanthamoeba* strains

*Acanthamoeba* were grown axenically and maintained in PYG (peptone yeast glucose, pH 6.5) [36]. All isolates were seeded in separate wells of 24-well culture plate with PYG medium (500 µL/well) and incubated statically at 28 °C until the trophozoites formed 90% confluent layers at the bottom of each well. Aliquots of the PYG were inoculated onto trypticase soy agar (Becton, Dickinson and Company, Sparks, MD, USA) and incubated at 37 °C for 48 h. At the end of the incubation, the agar plates were examined for growth of any bacteria. Before collection of trophozoites, PYG medium was gently replaced by 1 mL of PBS containing gentamicin (100 μg/mL) to kill any extracellular bacteria and the plate was put on ice with gentle agitation to dislodge adhered trophozoites. TRIzol reagent (Invitrogen, Life Technologies, New York, USA) was used for the whole gDNA extraction following the phenol–chloroform separation method as per manufacturer’s protocol. The method was slightly modified to exclude the addition of EDTA which can interfere with PCR assay by sequestering Mg^2+^ ions [37]. To enhance the recovery of gDNA, TRIzol-treated cell suspensions were passed 10 times through 25G syringe needles (BD, New Jersey, USA) to lyse the amoebal cells.

The 16S rRNA gene (V4 region) of intracellular bacteria was amplified using eubacteria 16S rRNA primer; 515Fw/806Rv: Fw (5′- CCTACGGGNGGCWGCAG-3′) and Rv (5’ – GACTACHVGGGTATCTAATCC-3′) [38]. Genomic DNA of *E. coli* ATCC 10,798 and nuclease free water were included as positive and negative controls, respectively. The presence of intracellular fungi was assessed using fungus-specific forward ITS1Fw (5’- TCCGTAGGTGAACCTGCGG-3’) and reverse ITS4Rv (5’-CCTCCGCTTATTGATATGC-3’) primers targeting the conserved sequences of 18S and 28S rDNAs [39]. A clinical isolate of *Candida albicans* was included as a control and intracellular fungus was identified by Sanger sequencing of PCR amplicon.

### Fluorescence in situ hybridization (FISH)

In order to visualise the intracellular bacteria or fungi, FISH in combination with confocal microscopy was performed following a previous protocol [40]. Briefly, 1 ml amoebal cells (> 90% trophozoites) grown axenically were collected in 1.5 ml eppendorf tubes and centrifuged for 5 min at 3,000 g to harvest trophozoites. The cell pellet was washed twice with 1X Page’s saline and 30µL of amoebic suspension was transferred on poly-l-lysine coated slides (Thermo Scientific, Braunschweig, Germany) and left for 30 min at ambient temperature. The adherent cells were fixed by applying 30µL of freshly prepared 4% formaldehyde (buffered, pH 6.9) for 25 min. The attached amoebal cells were washed with 1X PBS, dehydrated in increasing ethanol concentrations (50%, 80%, and 96%, 3 min each) and air-dried. Intracellular bacteria were examined by hybridization using Cy3 labelled bacterial-domain specific probe EUB338 [41], and fungi using the fungus-specific probe PF2 conjugated with Hex and a Cy5 labelled EUK516 probe (**Table ****1**) [42] for eukaryotic 18S rRNA (Biomers, Ulm, Germany). Aliquots (1µL of 50 ng/µL) of each probe were mixed with 9µL of hybridization buffer (20 mM Tris–HCl, pH 7.1, 900 mM NaCl, and 20% v/v formamide, 0.01% SDS) and added to the fixed amoebal cells on slides. Hybridization was carried out for at least 90 min at 46 °C in the dark after which slides were rinsed with 20µL of pre-warmed (48 °C) buffer (180 mM NaCl, 20 mM Tris/HCl, pH 7.2, and 0.01% SDS). The slides were then covered with 200µL buffer and a washing step was performed at 48 °C for 25 min. All slides were quickly immersed in ice-cold MilliQ water, air dried, and were mounted using Prolong Diamond Antifade with DAPI (Thermo Fisher Scientific), then mounted slides were left overnight to cure at room temperature in the dark before imaging. Three independent assays were performed and at least 30 amoebal host cells were visualized under Olympus FV1200 confocal laser scanning microscope in Katharina Gaus Light Microscopy Facility of UNSW and FISH images were analysed in ImageJ [43].

**Table 1:** FISH probe bases used in this study (probebase.csb.univie.ac.at )

Probe	Sequence (5′ → 3′)	Modification at 5′	Specificity	rRNA position	Refs.
EUB338	GCTGCCTCCCGTAGGAGT	Cy3	Most eubacteria	338–355 (16S)	[41]
PF2	CTCTGGCTTCACCCTATTC	Hex	Most yeasts	18/618 (18S)	[44]
EUK516	ACCAGACTTGCCCTCC	Cy5	Eukaryota	502–517 (18S)	[41]

### Clinical data

Demographic and clinical features of all 13 AK patients were retrieved in a customised datasheet from hospital records and were reviewed for the following features: initial and final visual acuity (VA); reported symptoms; duration, size and characteristics of infiltrates and epithelial defects (some measurements were transcribed from clinical images); prior treatment (defined as medicines used before the first presentation of patients at LVPEI); clinical treatment regimens and duration under care at LVPEI; need for surgery; treatment outcome; patients’ occupation; and reported risk factors for AK [45].

### Data presentation and statistical analysis

Data analysis was performed in SPSS software version 26.0 (SPSS, Inc., Chicago, IL). Proportions were presented as percentage and mean ± standard deviation was calculated for continuous data. A 95% confidence interval (CI) was calculated for demographic and clinical data using proportion (p) ± 1.96* SEM (standard error of mean). Fisher’s exact and chi square were used for comparison of demographic and clinical data with endosymbiont status with the level of significance of *P* value < 0.05 for 2-tailed tests.

## Results

**Fig. 1:**
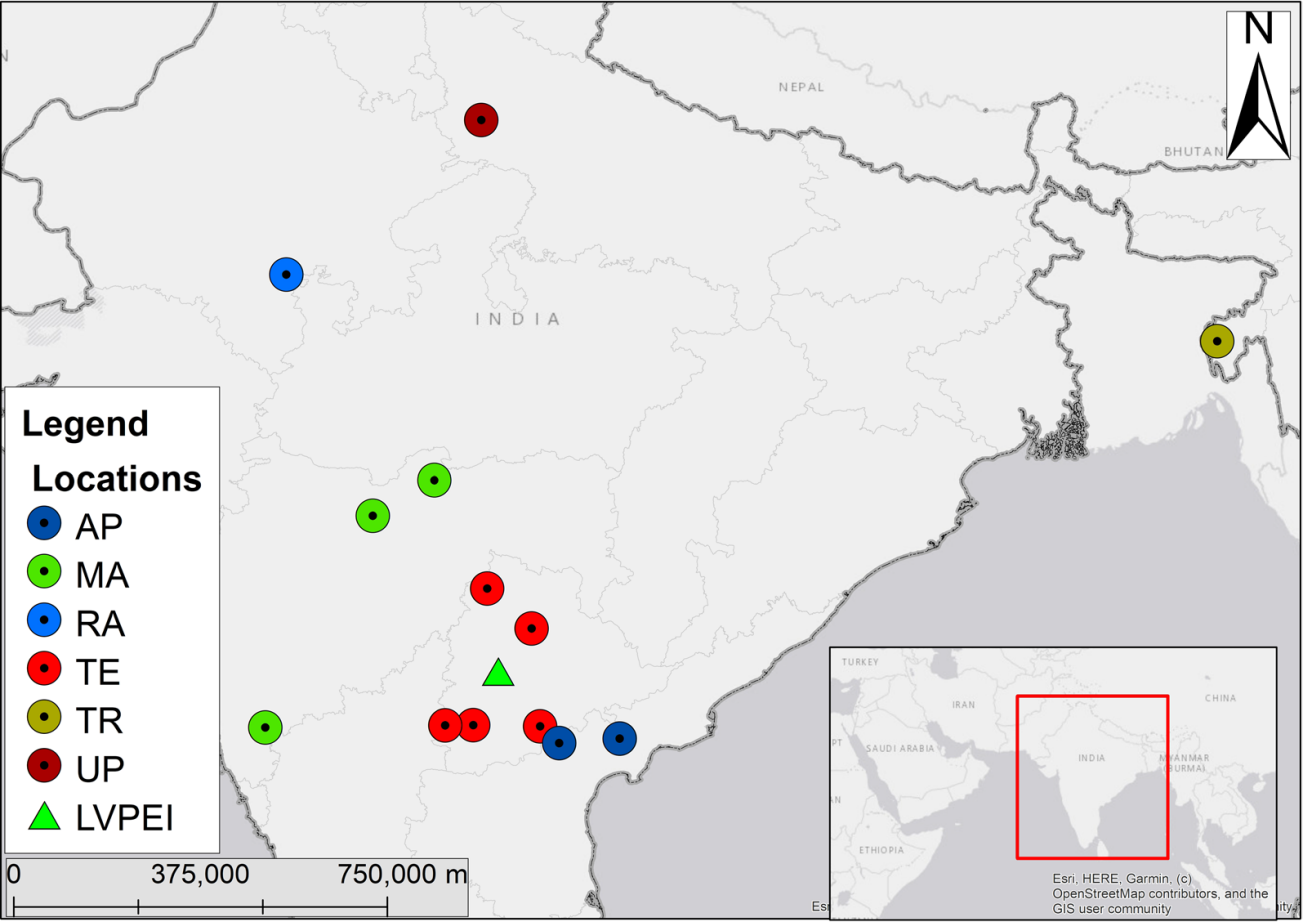
Map showing the states of the AK patients and LVPEI hospital in India. The map was created using ArcGIS (Esri GIS, California, USA). *AP* Andhra Pradesh, *MA* Maharashtra, *RA* Rajasthan, *TE* Telangana, *TR* Tripura, *UP* Uttar Pradesh

Among the 13 AK patients, the highest number of cases (30.8%, 4 AK patients) were observed in February (Additional file 1: Fig. S1). Patients were from six different states of India; five were from Telangana, three from Maharashtra, two from Andhra Pradesh, and one each from Rajasthan, Uttar Pradesh, and Tripura (Fig. 1).

### Isolation and identification of *Acanthamoeba* spp.

**Fig. 2:**
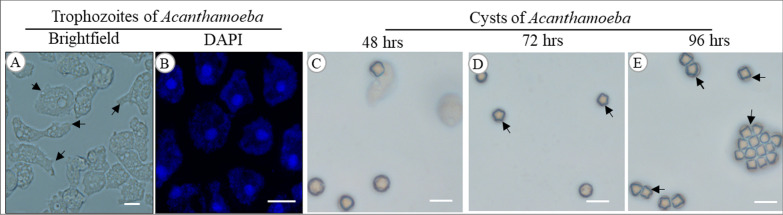
Structures of *Acanthamoeba* trophozoites (**A**) with acanthopodia, arrows indicate needle like projections on cell surface. **B**, confocal image of trophozoites’ nuclei stained with DAPI. **C**, **D**, phases of encystment of strain L-1326/20 from pre-cyst (**C**, **D**) to double-walled polygonal cysts (**E**), arrows indicate polygonal *Acanthamoeba* cysts. Scale bar, 10 µm

**Fig. 3:**
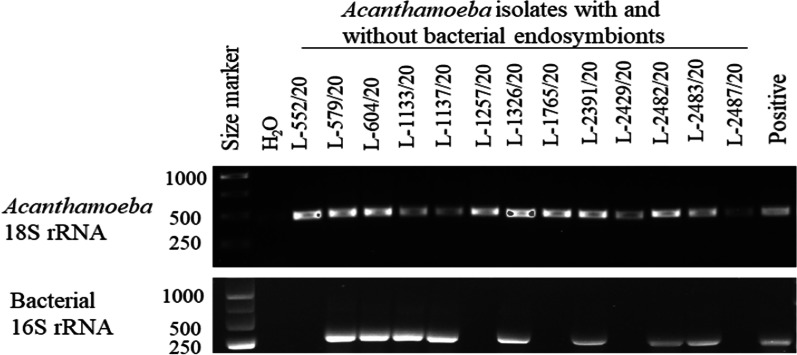
Cropped agarose gel image of PCR amplicons of *Acanthamoeba* isolates and intracellular bacteria. Bands were visualised using 1% gel electrophoresis; primer pairs JDPFw/Rv and 515Fw/806Rv yielded ~ 450 bp and ~ 293 bp amplicons, respectively. *A. castellanii* (ATCC 30868) and *E. coli* (ATCC 10798) were used as positive control for 18S rRNA and 16S rRNA PCR reactions and nuclease free water was used as negative control. Full size gel images are included in Additional file 1 (Figs. 4 and 5)

Identification of the *Acanthamoeba* strains and their growth at different temperatures are shown in Table 2. Seven isolates (53.8%) grew at 40 °C and 42 °C, demonstrating the thermotolerance of certain strains that cause AK. Figure 2 shows the trophozoites (1A and 1B) and cysts (1C-1E) of an *Acanthamoeba* isolate (L-2483/20). Figure 3 shows the agarose gel image of the PCR products of the DF3 region of amoebal 18S rDNA gene and the bacterial 16S rRNA. All amoebae were confirmed as being *Acanthamoeba,* of which eight (61.5%, 8/13) harboured intracellular bacteria and one isolate (L-2429/20) contained fungus. No bacteria grew from the PYG aliquots cultured on trypticase soy agar, suggesting that any bacteria identified in FISH experiments were very likely to be located intracellularly.

**Table 2:** List of *Acanthamoeba* isolates recovered in this study, GenBank nucleotide sequence accession numbers, and growth at different culture temperatures

S.N	Isolate ID	*Rns* Genotype	Species	GenBank accession no	Growth at different temperature (°C)			
					28	37	40	42
1	L-552/20	T4B	*A. culbertsoni*	OK042094	** + **	** + **	−	−
2	L-579/20	T4B	*A. polyphaga*	OK042095	** + **	** + **	** + **	** + **
3	L-604/20	T4B	*Acanthamoeba* spp.	OK042096	** + **	** + **	** + **	** + **
4	L-1133/20	T4B	*A. culbertsoni*	OK042097	** + **	** + **	−	−
5	L-1137/20	T4F	*A. triangularis*	OK042098	** + **	** + **	** + **	** + **
6	L-1257/20	T4B	*A. culbertsoni*	OK042099	** + **	** + **	−	−
7	L-1326/20	T4B	*A. polyphaga*	OK042100	** + **	** + **	−	−
8	L-1765/20	T4A	*Acanthamoeba* spp.	OK042101	** + **	** + **	−	−
9	L-2391/20	T12	*A. healyi*	OK042102	** + **	** + **	** + **	** + **
10	L-2429/20	T4B	*A. triangularis*	OK042103	** + **	** + **	−	−
11	L-2482/20	T4B	*A. culbertsoni*	OK042104	** + **	** + **	** + **	** + **
12	L-2483/20	T4B	*A. culbertsoni*	OK042105	** + **	** + **	** + **	** + **
13	L-2487/20	T4B	*A. culbertsoni*	OK042106	** + **	** + **	** + **	** + **

“ + ” denotes growth and “−” denotes no growth

**Fig. 4:**
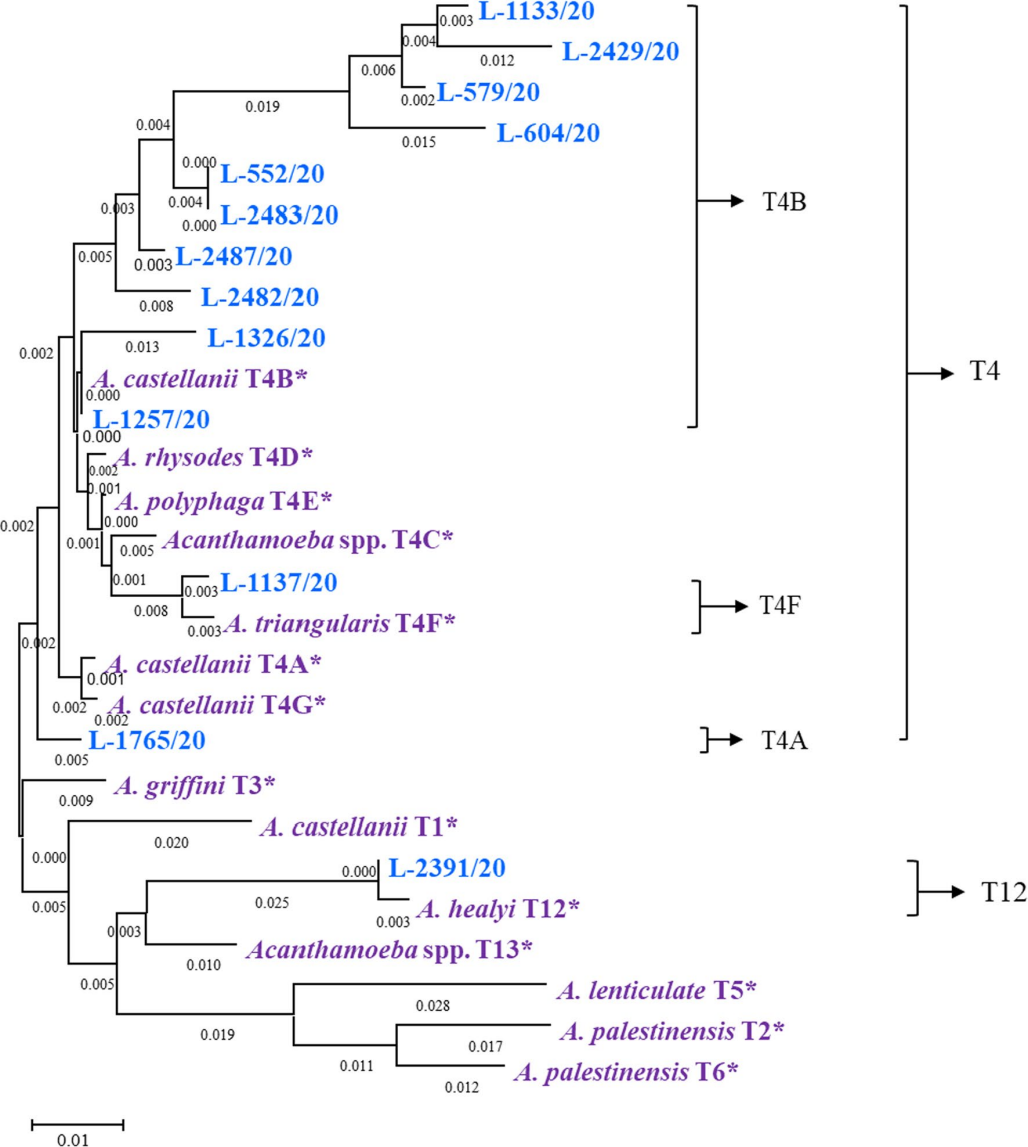
Phylogenetic tree inferred from the 18S rRNA sequences of *Acanthamoeba* isolates. The tree was created using the neighbour-joining approach with the Kimura 2-parameter based on 1,000 replicate bootstrap values. *Acanthamoeba* isolates (blue coloured) of this study formed two major genotypic clades; T4 was the predominant genotype (three sub-clusters: T4A, T4B, and T4F) and T12 had only one isolate, “*”denotes NCBI reference species and genotypes (purple coloured)

**Fig. 5:**
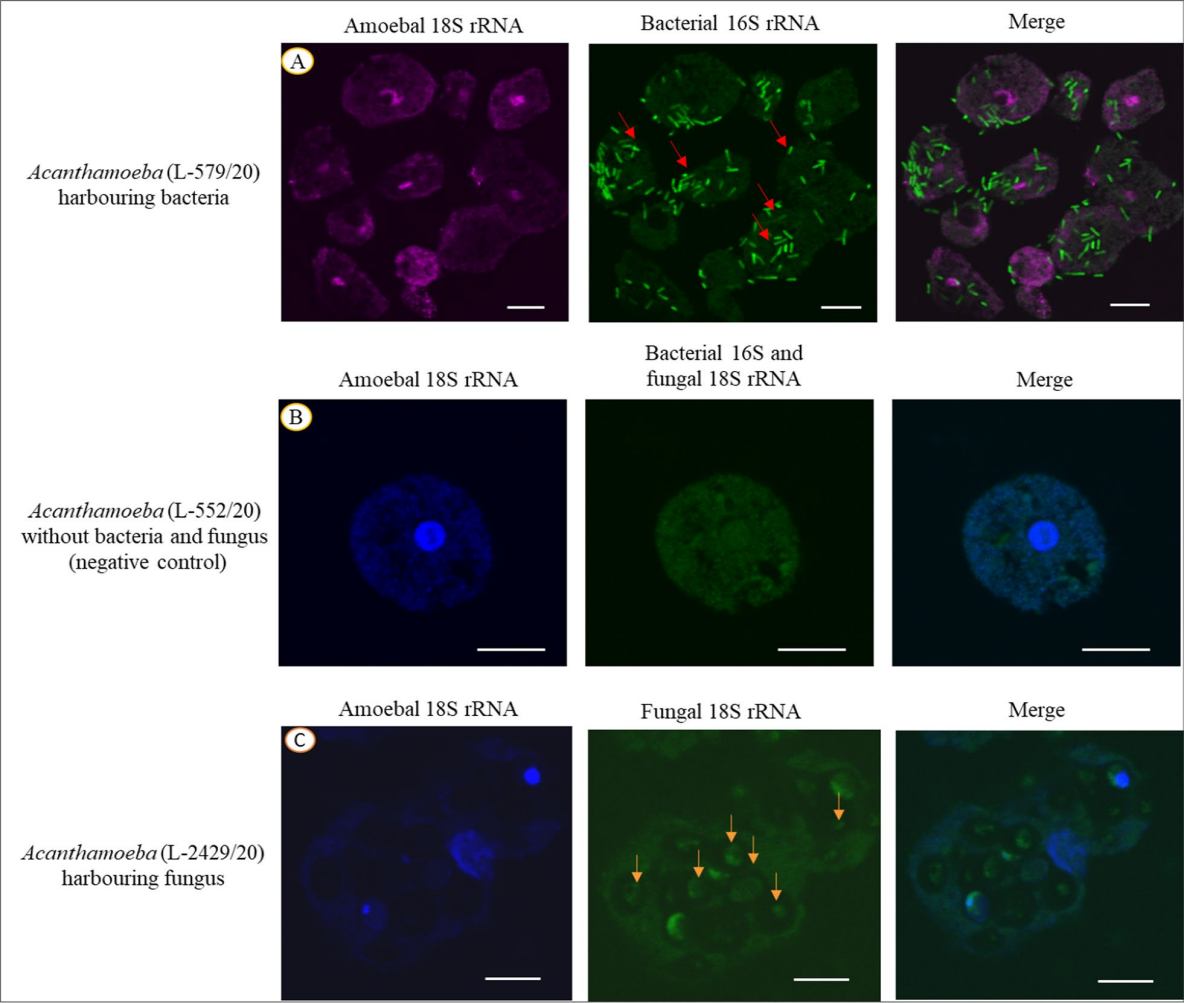
Representative images of FISH assay depicting intracellular bacteria **A** and fungi **C** of *Acanthamoeba* isolates. **A** Rod-shaped bacteria were dispersed throughout all the amoebal cells in the population which was detected using EUB338 probe (L-579/20, indicated by red arrows). **B** *Acanthamoeba* trophozoite (L-552/20) without intracellular bacteria or fungi. **C** Large ovoid fungal cells were observed within *Acanthamoeba* strain using PF2 probe (L-2429/20, indicated by yellow arrows). Scale bar, 12 µm

The partial nucleotide sequence of amoebal 18S rDNA DF3 region was aligned using ClustalW algorithm and showed the highest inter-strain nucleotide sequence variation (Additional file 1: Fig. S2). The neighbour-joining phylogenetic analysis of 13 isolates formed two major clades, with most isolates (12/13, 92.3%) belonging to genotype T4 (L-552/20, L-1133/20, L-1257/20, L-2482/20, L-2483/20, L-2487/20: *A. culbertsoni*; L-579/20, L-1326/20: *A. polyphaga*; L-1137/20, L-2429/20: *A. triangularis*, and L-604/20, L-1765/20: *Acanthamoeba* spp. T4) and one isolate (L-2391/20) being genotype T12 (*A. healyi*; Table 2 and Additional file 1: Table S2). Along with cyst morphology, the sequence producing the highest alignment (% identity) with sequences in NCBI of existing species and genotypes of *Acanthamoeba* was assigned to each isolate. For two isolates (L-604/20 and L-1765/20), cyst morphology was not obvious to a particular species of *Acanthamoeba* but the NCBI nucleotide blasting, and phylogenetic analysis confirmed both strains belonged to genotype T4. Genotype T12 was detected from a farmer from Maharashtra with no history of ocular trauma; two other cases from the same state were infected with genotype T4. The remaining ten isolates of T4 variants were recovered from AK patients from Andhra Pradesh, Rajasthan, Telangana, Tripura, and Uttar Pradesh (Additional file 1: Map S1). In the predominant T4 clade, the isolates formed three sub-clusters, T4A (1 isolate), T4B (10 isolates), and T4F (1 isolate). The nucleotide sequence of all 13 isolates have been deposited at GenBank under accession numbers OK042094 to OK042106 (Table 2). Of the genotype T4B, 6 patients were infected with *A. culbertsoni*, 2 with *A. polyphaga*, and one each with *A. triangularis* or *Acanthamoeba* spp. One patient was infected with T12 *A. healyi* and another patient (L-1765/20) was with T4A *Acanthamoeba* spp. (Fig. 4)*.*

### Endosymbionts in *Acanthamoeba* isolates recovered from AK patients

Among the 13 *Acanthamoeba* isolates, 9 (69.2%) were positive for intracellular microbes with eight harbouring bacteria (Fig. 3) and one isolate (L-2429/20) harbouring the fungus *Malassezia restricta* (Additional file 1: Fig. S3). For the eight isolates harbouring bacteria, rod-shaped intracellular bacteria were observed throughout the cytoplasm, and the bacteria were present in all *Acanthamoeba* cells in the population observed. No extracellular bacteria were observed, as expected due to the protocol. *M. restricta* was seen as oval-shaped green cells within *Acanthamoeba* isolate (Fig. 5).

The mean symptom duration of AK patients infected by *Acanthamoeba* with intracellular bacteria or fungus was 43.0 ± 44.3 days compared to 16.5 ± 5.1 days of patients infected with endosymbiont free *Acanthamoeba* (Table 3). Among those nine AK cases, six had a history of either ocular trauma (*n* = 4) or contact lens wear (*n* = 2) and three were farmers. A severe form of AK with stromal infiltrate (88.9%, 8/9) and hypopyon (55.6%, 5/9) was noted in the presence of amoebal endosymbionts and five cases had both stromal infiltrate and hypopyon. However, these differences in stromal infiltrate (*p* = 0.2) and hypopyon (*p* = 1.0) were not significantly more common in AK patients with amoebal endosymbionts compared to those without the endosymbionts. Interestingly, a longer medical treatment duration (median, IQR: 75.0, 43.5–202.5 vs. 30.0, 17.0–91.8 days) was observed in patients without *Acanthamoeba* endosymbionts (*p* = 0.2). In the presence of endosymbionts, the proportion of patients with a healing ulcer (66.6%) or an improvement in final VA (44.4%) were lower compared to those without endosymbionts (75% and 50%, respectively) (*p* > 0.05). Two patients (25%, 2/8) infected by *Acanthamoeba* with bacterial endosymbionts had received antibiotics along with antiamoebic drugs and the ulcers of both cases were resolved after medication.

**Table 3:** Comparison of AK patient outcomes with respect to *Acanthamoeba* with and without endosymbionts

S. No.	Clinical features	*Acanthamoeba* with endosymbionts (*n* = 9)	*Acanthamoeba* without endosymbionts (*n* = 4)
1	Symptom duration at presentation, days (mean ± SD)	43.0 ± 44.3	16.5 ± 5.1
2	Reported risk factor (trauma and/or contact lens wear)	55% (5/9)	25% (1/4)
3	Observation of stromal infiltrate	88.9% (8/9)	50% (2/4)
4	Presence of hypopyon	55.6% (5/9)	25% (1/4)
5	Medical treatment duration, days (median, IQR)	30.0 (17.0–91.8)^§^	75.0 (43.5–202.5)
6	Healing of ulcer	66.6% (6/9)	75% (3/4)
7	Improved final VA by ≥ 2 lines	44.4% (4/9)	50% (2/4)

Data are expressed as mean ± standard error of the mean, *VA* visual acuity, ^§^excluding a case with a treatment duration of 5 days

### Clinical characteristics of AK patients

Most of the AK patients were farmers (5/13) or students (5/13) and the reported risk factors associated with AK were ocular trauma (4 cases; 30.8%; 95% CI = 9.1—61.4) and contact lens wear (2 cases; 15.4%; 95% CI = 1.9—45.5). Unilateral AK was reported in 12 (92.3%) cases. The majority of AK patients had decreased vision (84.6%) as a major symptom followed by eye pain (69.2%), redness (69.2%), watering (53.8%), and white spot on the cornea (15.4%). A typical AK characteristic ring infiltrate (> 4 mm) was observed in 3 (23.1%) cases. Epithelial defect was noted in 6 cases (46.2%; 6.2 ± 1.7 mm), stromal infiltrates in 10 cases (76.9%; 5.0 ± 2.2 mm), and hypopyon in 6 cases (46.1%; 1.12 ± 0.6 mm). The median duration of symptoms onset was 20 days (IQR = 15—30) and the final visual acuity was not improved by ≥ 2 lines among patients with farming background (5/5, 100%), age > 32 years (4/6, 80%) and patients showing AK symptoms more than 20 days (3/6, 50%) (Additional file 1: Table S3). PHMB and chlorohexidine were the most common treatments in this study (11 cases; 84.6%). Three cases received antibiotics, one case antiviral and one case antifungal as supportive therapy (38.5%); for these there was no improvement (*p* > 0.05) in BCVA at final presentation compared to cases treated only with PHMB and chlorohexidine. Overall, the median duration of medical treatment was 38 days (IQR = 23—90). Of six cases with surgical treatment, 4 cases had therapeutic penetrating keratoplasty (TPK, *n* = 4), including one case that had an amniotic membrane transplant (Table 4). Of the remaining two cases, one had photodynamic antimicrobial therapy with rose bengal (RB-PDAT) and one underwent evisceration. Among 6 patients who had to undergo ocular surgery, 66.7% (4/6) were infected by *Acanthamoeba* strains with intracellular bacteria.

**Table 4:** Clinical characteristic AK patients with respect to the *Acanthamoeba* genotype and its species

S.N	Sample ID	Patients age (years), gender, occupation	History of Ocular trauma, CL (yes/no)	Stromal infiltrate	Hypopyon	Major symptoms	Size of epithelial defect (vxh) mm	Medical treatment duration (days)	Surgical treatment	Healing of ulcer	Species, genotype	Presence or absence of endosymbionts
1	L-552/20	33, M, Farmer	NK, no	Yes	No	Pain, redness	0	38	No	Healed	*A. culbertsoni*, T4B	None detected
2	L-579/20	25, M, Student	NK, no	Yes	Yes	Pain, FB sensation, watering, redness	4 × 4.5	420	TPK, TABCL	Improved, s/p TPK	*A. polyphaga*, T4B	Bacteria
3	L-604/20	8, F, Student	NK, no	Yes	No	Pain, watering, redness, decrease vision	6 × 6	5	No	Improved	*Acanthamoeba* spp., T4B	Bacteria
4	L-1133/20	32, F, Mason	NK, no	Yes	Yes	Pain, watering, decrease vision	4.5 × 4.5	23	No	Non-healing	*A. culbertsoni*, T4B	Bacteria
5	L-1137/20	56, M, Farmer	Ocular injury, no	Yes	Yes	Pain, redness, decrease vision	5.5 × 7	100	TPK, TABCL, AMG	Improved	*A. triangularis*, T4F	Bacteria
6	L-1257/20	43, M, Business	NK, no	No	No	Pain, redness	NK	240		Improved, s/p TPK	*A. culbertsoni,* T4B	None detected
7	L-1326/20	37, M, Business	NK, yes	Yes	No	Decrease vision, redness, whitish spot on cornea	NK	15	No	Improved	*A. polyphaga*, T4B	Bacteria
8	L-1765/20	14, F, Student	NK, no	No	Yes	Pain, watering, redness, whitish spot on cornea	NK	90	TPK	Improved, s/p TPK	*Acanthamoeba* spp., T4A	None detected
9	L-2391/20	20, F, Student	NK, no	Yes	Yes	Watering, decrease vision, redness, whitish spot on cornea	7.2 × 9	67	RB-PDAT	Improved	*A. healyi*, T12	Bacteria
10	L-2429/20	25, M, Farmer	Chemical injury, no	No	No	Pain, redness, decrease vision	Perforated cornea	11	No	Improved	*A. triangularis,* T4B	Fungi
11	L-2482/20	42, M, Farmer	Ocular injury, no	Yes	Yes	Redness, decrease vision	Tissue adhesive was in place	29	Tarso, AMG, TPK (post-injury)	Non-healing	*A. culbertsoni*, T4B	Bacteria
12	L-2483/20	22, F, Student	NK, yes	Yes	No	Watering, redness, decrease vision	NK	30	TPK	Improved	*A. culbertsoni*, T4B	Bacteria
13	L-2487/20	72, M, Farmer	Ocular injury, no	Yes	No	Pain, FB sensation, watering, redness	6 × 6	60	Evisceration	Worsened	*A. culbertsoni*, T4B	None detected

*M* male, *F* female, *CL* contact lens, *FB* foreign body, *NK* not known/reported, *TPK* therapeutic penetrating keratoplasty, *TABCL* Cyanoacrylate tissue adhesive with BCL (bandage contact lens), *AMG* amniotic membrane graft, *s/p* status post, *RB-PDAT* Rose Bengal-photodynamic antimicrobial therapy

## Discussion

Thirteen clinical isolates of *Acanthamoeba* spp. from Hyderabad, India were genotyped and clinical presentations associated with AK patients were analysed. T4 was the most common genotype (92.3%), as has been reported globally [45–48]. *A. culbertsoni* (6, 46.2%) was the predominant species among the T4 genotype, and this has been previously reported in India [45]. One isolate recovered from a patient with no pre-existing risk factors belonged to clade T12, which has infrequently been isolated from AK cases [19].

The current study supports that the predominant keratitis causing T4 genotype is also prevalent in multiple states (Andhra Pradesh, Maharashtra, Rajasthan, Telangana, Tripura and Uttar Pradesh) of India. To the best of the authors’ knowledge, this is only the second report of *A. healyi* T12 genotype from corneal infection in India [45]. A severe AK case caused by genotype T12 has been reported from Bangkok, Thailand [19] where the patient had history of exposure to a corrosive chemical and had used tap water to rinse infected eye. Among the T4 strains (92.3%, 12/13), the majority were T4B (83.3%, 10/12), with one isolate each of T4A and T4F. A previous Indian study reported 13 strains (50%) of the T4B sub-cluster from 26 isolates with others being T4A (two isolates), T4D (10 isolates), and T4E (one isolate) [45]. T4A (38%) was the leading sub-genotype of *Acanthamoeba* isolates recovered from Chilean AK patients, followed by T4B, T4G, T4C and T4D [46]. Among T4 strains, other DF3 variants such as T4E, F, G, I, J, N, O, P, V and X associated with corneal infections have been reported from different countries [46, 49, 50]. These findings imply a geographic difference among T4 isolates causing AK but this requires further investigation.

The incidence of AK is steadily increasing worldwide with approximately 90% of cases associated with contact lenses in developed countries [49]. Although contact lens wear is not commonly associated with AK in India and other developing nations, [45, 51] the global surge of myopia and the use of contact lens for preventing myopia, cosmetic purposes and sport activities have increased the risk of AK, especially among youths [1]. As observed in the current study, ocular trauma and contact with contaminated soil or water are the major predisposing risk factors of AK infection not linked with contact lens wear [52–54]. Ocular injury with either dust particles or vegetative matters was the most common reported risk factor among AK patients in central China (52.1%) [55] and south India (48.7%) [52].

The final VA was not improved by 2 or more lines among farmers (100%), patients aged > 32 years (80%) and cases showing keratitis symptoms for > 20 days (50%). A study from the UK has also reported worst clinical outcomes for AK patients aged > 34 years [56]. Corneal integrity weakens with age and the higher incidence of dry eyes among older populations may be possible predisposing factor for severe form of AK [9]. A previous study of bacterial keratitis from LVPEI supports the association of epithelial defect size with VA loss found in the current study [57]. Patients’ professions are often linked with the risk factors [55]. In the current study, among five cases having farming background, four (80%) had history of ocular trauma which may have been caused by non-sterile external matters as framers are frequently exposed to outdoor activities and three patients (75%, 3/5) were infected by *Acanthamoeba* strains with intracellular bacteria. All four cases had to undergo ocular surgery but visual acuity was not improved even after postoperative recovery. Ocular trauma with contaminated objects may be an ideal vehicle for invasion of *Acanthamoeba* cells leading to severe form of AK. Approximately 90% of AK patients with history of eye injury had corneal grafts to restore vision in Southern China [58].

Among the 13 *Acanthamoeba* isolates, 8 (61.5%) possessed intracellular bacteria and this is very similar to the 59.4% of *Acanthamoeba* possessing intracellular bacteria in a study from the USA [25], but greater by two to three fold than strains in the American Type Culture collection [26] or an older study (1993) from the USA that used only traditional staining techniques to disclose the intracellular microbes [59]. Intracellular bacteria belonging to the genera *Pseudomonas, Legionella, Chlamydia*, and *Mycobacterium* have been detected in *Acanthamoeba* isolates recovered from AK cases in the USA [25] while Rickettsiales, *Mycobacterium* and *Parachlamydia* spp. were detected in ATCC strains isolated from human nasal swab, cornea, and lake sediment, respectively [26]. Fritsche et al. [59] have observed rod and cocci shaped bacteria in both environmental (24%) and clinical (26%) strains of *Acanthamoeba* from different locations. Co-occurrence of phylogenetically diverse intracellular microbes within a *Acanthamoeba* cell has been observed [60]; *Aspergillus* spp., *P. aeruginosa,* and HAdV (human adenovirus) were detected in a clinical isolate of *Acanthamoeba* in Iran [61]. To the best of our knowledge, this is the first report describing *M. restricta* as an endosymbiont of *Acanthamoeba*. The patient was a farmer with a history of eye trauma with a chemical agent and the corneal ulcer was not resolved with medical treatment using PHMB and chlorhexidine for a month. *Acanthamoeba* and *Malassezia* coinfection has not been described in AK patients but *M. restricta* has infrequently caused fungal keratitis [62]. Other studies have reported keratitis due to co-infection of *Acanthamoeba* with fungi such as *Cladosporium*, *Fusarium* and *Curvularia* [23, 63, 64].

Intracellular bacteria of *Acanthamoeba* may enhance corneal epithelial damage as has been shown in a clinical study and a cell model [25]. Although in the current study there were no significant differences in clinical manifestations, the presence of bacterial endosymbionts was associated with a higher proportion of stromal infiltrates (87.5%), epithelial defects (62.5%) and hypopyon (50%). Based upon the current data, to show significant effect of these differences a minimum of 54 subjects (z-test for the difference between two independent proportions; α error = 0.05, 1-β error = 0.8, z score = 1.95 and allocation ratio = 1:1) is required in future studies (G*Power v3.1.9.7).

In the current study, two cases (25%, 2/8) affected by *Acanthamoeba* with bacterial endosymbionts had received antibiotics (ciprofloxacin and doxycycline) along with topical antiamoebic drugs and the ulcer of both cases resolved after treatment. The remaining cases of AK with intracellular bacteria healed when treated only with PHMB and chlorhexidine. This is expected as these antiseptics are also effective against bacteria [65]. Addition of antibiotics may be advantageous to antiamoebic chemotherapy to abrogate virulence-enhancing traits of intracellular bacteria [26]. On the other hand, release of endosymbionts after the death of amoebal host in a compromised cornea may boost inflammation and may worsen the clinical outcomes. At present, it is not fully understood whether auxiliary remedies to anti-amoebic treatment such as antibiotics are effective compared with anti‐amoebic drugs alone [66] and the role of endosymbionts during amoebal infections has not been fully explored yet [25]. The sample size of the current study may be inadequate to gauge clinically significant differences among these two groups. Yet as a baseline study with pilot data, this study is valuable for epidemiological and taxonomic purposes of *Acanthamoeba* keratitis. Future studies are necessary to examine the impact of amoebic endosymbionts on host pathogenicity, drug susceptibility, clinical outcome, and benefits of adding antibacterial adjuvants to standard antiamoebic chemotherapy with higher cohort and extended follow-up of AK patients.

## Conclusions

This study confirms that the keratitis causing *Acanthamoeba* isolates were mainly of T4 genotype followed by T12, with three sub-genomic variants T4A, T4B, and T4F identified within the predominant T4 genotype. There was no significant association between *Acanthamoeba* species and/or genotypes and a patient’s clinical outcome. In the current study, a high number of *Acanthamoeba* strains with endosymbionts were observed and although there was no clear relationship with clinical outcome, *Acanthamoeba* endosymbionts may be involved in shaping virulence, survival, and drug susceptibility of amoebal host.

## Supplementary Information

**Additional file 1: Table S1:** Reference *Acanthamoeba *strains used in this study for phylogenetic analysis. **Table 2:** Genotype and species identification of Acanthamoeba isolates recovered from AK patients. **Map 1:** Map showing the states of the AK patients from different states of India with sample codes and identified genotypes of Acanthamoeba from AK patients. The map was created using ArcGIS (Esri GIS, California, USA). **Fig. S1.** Monthly distribution of AK cases during study period. **Fig. S2.** Sequence alignment of *Acanthamoeba* 18S rDNA DF3 region using ClustalW. **Table S3.** Overall clinical presentation of the keratitis patients infected with *Acanthamoeba* spp. **Fig S3. **Phylogenetic tree inferred from the 18S (ITS1) rDNA sequence of fungi; tree was created using the neighbour-joining approach with the Kimura 2-parameter based on 1000 replicates bootstrap values. **Fig. S4. **Agarose (1%) gel image of PCR amplicons of 13 *Acanthamoeba *isolates (18S rRNA), PCR assay was performed using *Acanthamoeba *genus specific primer pair JDPFw and JDPRv which yielded ~450bp amplicons. **Fig. S5: **Agarose (1%) gel image of PCR amplicons of 13 *Acanthamoeba* isolates targeting intracellular bacteria 16S rRNA, primer pair 515Fw and 806Rv (V4, 16S rRNA) was used which yielded ~293bp amplicons.

## Data Availability

The clinical data of AK patients is available in excel and SPSS sheets which can be obtained from the corresponding author on reasonable request. The assigned GenBank accession number of the nucleotide sequence ranged from OK042094 to OK042106 (https://www.ncbi.nlm.nih.gov/nuccore/?term=OK042094:OK042106[accn]).

## Abbreviations

AK: *Acanthamoeba* Keratitis
ASA.S1: *Acanthamoeba* Specific amplimer
BCVA: Best-corrected visual acuity
dNTP: Deoxynucleoside triphosphate
DF3: Diagnostic fragment 3
LVPEI: L. V. Prasad Eye Institute
PHMB: Polyhexamethylene biguanide
*Rns*: Nuclear small subunit 18S ribosomal RNA gene
NNA: Non-nutrient agar
FISH: Fluorescence in situ hybridization
PCR: Polymerase chain reaction
RB-PDAT: Rose bengal photodynamic antimicrobial therapy
