# Supplementary material for: Assessment of genotypes, endosymbionts and clinical characteristics of Acanthamoeba recovered from ocular infection
Source: BMC Infect Dis. 2022 Sep 29;22:757. doi: 10.1186/s12879-022-07741-4 (PMC9520893; doi:10.1186/s12879-022-07741-4)
Supplement: Supplementary file 1 — Additional file 1: Table S1: Reference Acanthamoeba strains used in this study for phylogenetic analysis. Table 2: Genotype and species identification of Acanthamoeba isolates recovered from AK patients. Map 1: Map showing the states of the AK patients from different states of India with sample codes and identified genotypes of Acanthamoeba from AK patients. The map was created using ArcGIS (Esri GIS, California, USA). Fig. S1. Monthly distribution of AK cases during study period. Fig. S2. Sequence alignment of Acanthamoeba 18S rDNA DF3 region using ClustalW. Table S3. Overall clinical presentation of the keratitis patients infected with Acanthamoeba spp. Fig S3. Phylogenetic tree inferred from the 18S (ITS1) rDNA sequence of fungi; tree was created using the neighbour-joining approach with the Kimura 2-parameter based on 1000 replicates bootstrap values. Fig. S4. Agarose (1%) gel image of PCR amplicons of 13 Acanthamoeba isolates (18S rRNA), PCR assay was performed using Acanthamoeba genus specific primer pair JDPFw and JDPRv which yielded ~450bp amplicons. Fig. S5: Agarose (1%) gel image of PCR amplicons of 13 Acanthamoeba isolates targeting intracellular bacteria 16S rRNA, primer pair 515Fw and 806Rv (V4, 16S rRNA) was used which yielded ~293bp amplicons. [file 12879_2022_7741_MOESM1_ESM.docx]

**Supplementary table 1:** Reference *Acanthamoeba* strains used in this study for phylogenetic analysis

| **S.N.** | ***Acanthamoeba* strain** | **ATCC** | **Genotype/sub-genotype** | **Accession number** |
| --- | --- | --- | --- | --- |
|  | *A. castellanii* | 50494 | T1 | U07400 |
|  | *A. palestinensis* | 30870 | T2 | U07411 |
|  | *A. griffin* | 30731 | T3 | U07412 |
|  | *A. castellanii* | 30011 | T4A | U07413 |
|  | *A. castellanii* | 50370 | T4B | U07414 |
|  | *Acanthamoeba* spp. | 50369 | T4C | U07409 |
|  | *A. rhysodes* | 30973 | T4D | AY351644 |
|  | *A. polyphaga* | 30871 | T4E | AF019061 |
|  | *A. triangularis* | 50254 | T4F | AF346662 |
|  | *A. castellanii* | 30010 | T4G | U07416 |
|  | *A. lenticulate* | 50428 | T5 | U94739 |
|  | *A. palestinensis* | 50708 | T6 | AF019063 |
|  | *A. healyi* | 30866 | T12 | AF019070 |
|  | *Acanthamoeba* spp. | PRA-3 | T13 | AF132134 |

**Supplementary FIG 1:** Monthly distribution of AK cases during study period


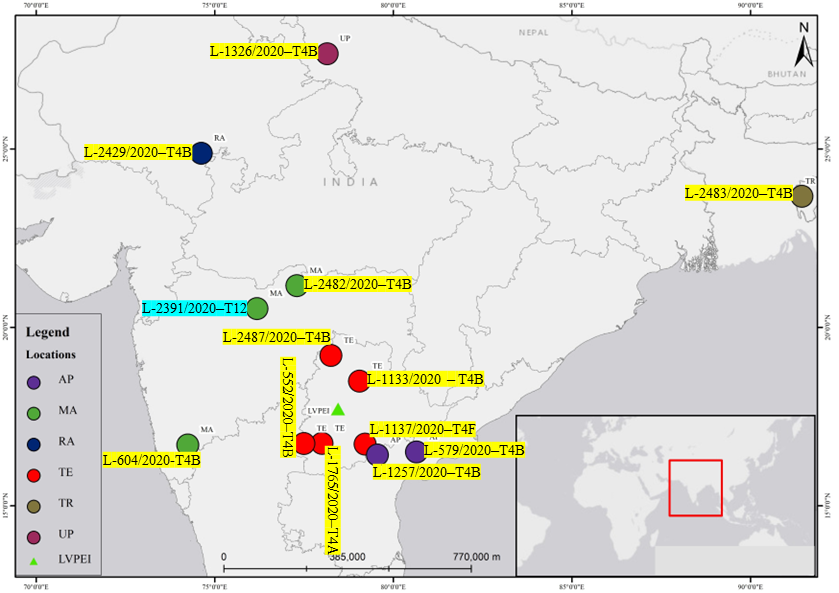


**Supplementary Map 1:** Map showing the states of the AK patients from different states of India with sample codes and identified genotypes of *Acanthamoeba* from AK patients. The map was created using ArcGIS (Esri GIS, California, USA)

**Supplementary FIG 2:** Sequence alignment of Acanthamoeba 18S rDNA DF3 region using ClustalW. Sequences represent a subset of the DF3 highly variable region.

Keys: “#” denotes NCBI reference strains, “*” represents similarities between all isolates and “-” gaps in nucleotide sequence

**Supplementary table 2:** Genotype and species identification of *Acanthamoeba* isolates recovered from AK patients

| **Sample ID** | **Primer** | **NCBI BLASTn result** | **Sequence identity of isolates to published strains (% identity, highest homology), accession number** |
| --- | --- | --- | --- |
| L-552 | JDP-Fw | *Acanthamoeba* T4 | *A. culbertsoni* (96.6%), MN091854 |
| L-579 | JDP-Fw | *Acanthamoeba* T4 | *A. polyphaga* (97.05%), KF881888 |
| L-604 | JDP-Fw | *Acanthamoeba* T4 | *Acanthamoeba* spp. T4 (100%), OK042096 |
| L-1133 | JDP-Fw | *Acanthamoeba* T4 | *A. culbertsoni* (97.6%), MH791023 |
| L-1137 | JDP-Fw | *Acanthamoeba* T4 | *A. triangularis* (98%), AF316547 |
| L-1257 | JDP-Fw | *Acanthamoeba* T4 | *A. culbertsoni* (99.8%), MN700306 |
| L-1326 | JDP-Fw | *Acanthamoeba* T4 | *A. polyphaga* (96.2%), MK713916 |
| L-1765 | JDP-Fw | *Acanthamoeba* T4 | *Acanthamoeba* spp. T4 (100%), OK042101 |
| L-2391 | JDP-Fw | *Acanthamoeba* T12 | *A. healyi* (98.8%), MK713915 |
| L-2429 | JDP-Fw | *Acanthamoeba* T4 | *A. triangularis* (96.1%), MN153012 |
| L-2482 | JDP-Fw | *Acanthamoeba* T4 | *A. culbertsoni* (96.9%), MN091854 |
| L-2483 | JDP-Fw | *Acanthamoeba* T4 | *A. culbertsoni* (96.5%), MN091854 |
| L-2487 | JDP-Fw | *Acanthamoeba* T4 | *A. culbertsoni* (97.5%), MN091854 |

**Supplementary table 3:** Overall clinical presentation of the keratitis patients infected with *Acanthamoeba* spp.

| **SN** | **Demographic and clinical features** | **Variables** | **Number of Patients (%)** |
| --- | --- | --- | --- |
|  |  |  |  |
| 1 | Gender | Male | 8 (61.5) |
|  |  | Female | 5 (38.5) |
| 2 | Overall median age = 32 years, (IQR, 21-42.5 years) | ≤ 32 years | 7 (53.8) |
|  |  | > 32 years | 6 (16.2) |
| 3 | Occupation | Farmer | 5 (38.5) |
|  |  | Student | 5 (38.5) |
|  |  | ^#^Other | 3 (23.0) |
| 4 | Laterality | Unilateral | 12 (92.3) |
|  |  | Bilateral | 1 (7.7) |
| 5 | Symptoms | Decreased vision | 11 (84.6) |
|  |  | Pain | 9 (69.2) |
|  |  | Redness | 9 (69.2) |
|  |  | Watering | 7 (53.8) |
|  |  | White spot on the cornea | 3 (15.4) |
| 6 | Number of symptoms | ≤ 3 symptoms  > symptoms | 8 (61.5)  5 (38.5) |
| 7 | Symptom duration  (Median = 20 days; IQR = 15 – 30) | ≤ 20 days | 7 (53.8) |
|  |  | > 20 days | 6 (46.2) |
| 8 | Aetiological factor | Present  (CL=2, trauma=4) | 6 (46.2) |
|  |  | Absent | 7 (53.8) |
| 9 | Epithelial defect | Yes | 6 (46.1) |
|  |  | No | 6 (46.1) |
|  |  | Missing data | 1 (7.7) |
| 10 | Stromal infiltrate | Yes | 10 (76.9) |
|  |  | No | 3 (23.1) |
| 11 | Ring infiltrates | Present | 3 (23.1) |
|  |  | Absent | 9 (69.2) |
|  |  | Missing data | 1 (7.7) |
| 12 | Hypopyon | Present | 6 (46.1) |
|  |  | Absent | 7 (53.8) |
| 13 | Prior treatment | Antibacterial/antifungal | 10 (76.9) |
|  |  | Others (antiviral, anti-*Acanthamoeba*) | 3 (23.1) |
| 14 | Medical treatment | PHMB/Chlorhexidine | 6 (46.2) |
|  |  | Antibiotics/Antifungal | 2 (15.4) |
|  |  | Combined | 5 (38.4) |
| 15 | Duration of medical treatment (Median = 38 days; IQR = 23-90) | ≤ 38 days | 7 (53.8) |
|  |  | > 38 days | 6 (46.2) |
| 16 | Surgery for ocular complication | Yes | 6 (46.2) |
|  |  | No | 7 (53.8) |
| 17 | Status of the ulcer at final hospital visit | Healed/improved | 10 (76.9) |
|  |  | Worse/not improved | 3 (23.1) |

CL = contact lens; # 3 cases (homemaker: 1, business: 2); ^§^Analysis by excluding missing data; *Fisher’s exact test; IQR = interquartile range, ^¶^ = Analysis by excluding a case with a treatment duration of 5 days.

In pre-treatment category, combined is represented by antibacterial and antifungal; anti-viral agent was added in one case along with bandage contact lens; surgery = photo-keratectomy therapy, amniotic membrane transplant and evisceration

**
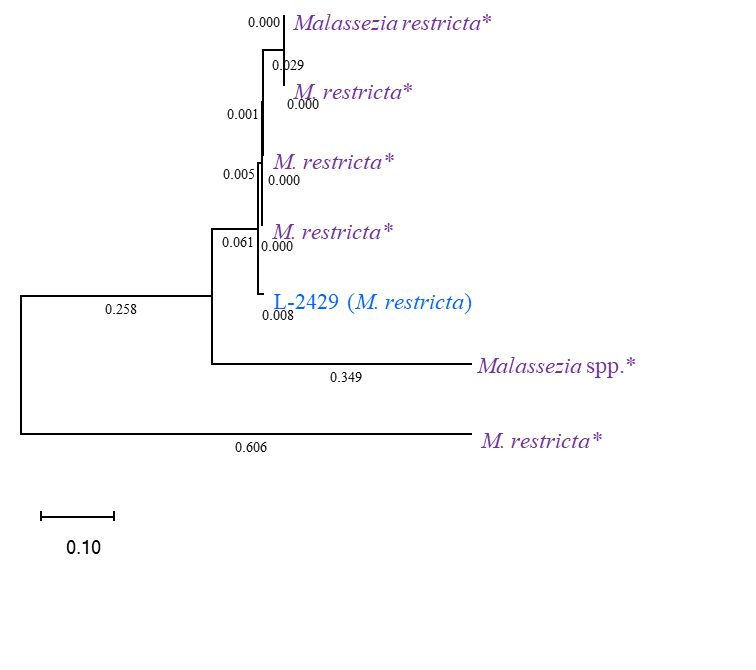
**

**Supplementary FIG 3:** Phylogenetic tree inferred from the 18S (ITS1) rDNA sequence of fungi; tree was created using the neighbour-joining approach with the Kimura 2-parameter based on 1,000 replicates bootstrap values. The intracellular fungus of *Acanthamoeba* isolate (L-2429) belonged to *Malassezia* spp. (indicated by blue colour), “*” denotes NCBI reference species and genotypes (indicated by purple colour).

**
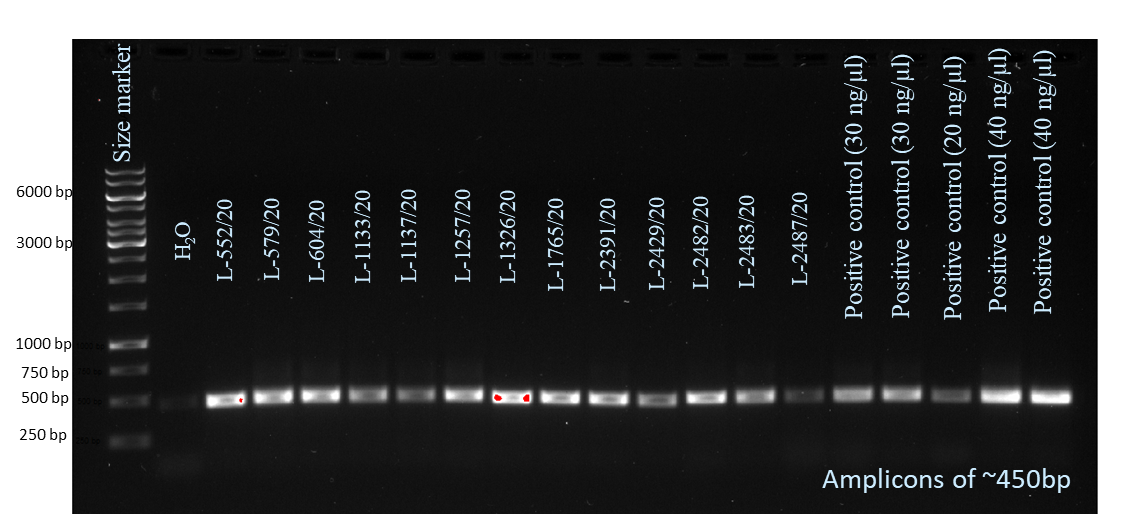
**

**Supplementary FIG 4:** Agarose (1%) gel image of PCR amplicons of 13 *Acanthamoeba* isolates (18S rRNA), PCR assay was performed using *Acanthamoeba* genus specific primer pair JDPFw and JDPRv which yielded ~450bp amplicons. *A. castellanii* (ATCC 30868) was used as positive control of different DNA concentrations (20, 30 and 40 ng/µL) to confirm the efficacy of PCR. Image was captured using Gel Doc XR^+^ with Image lab Software (Bio Rad Laboratories, California, USA).

**
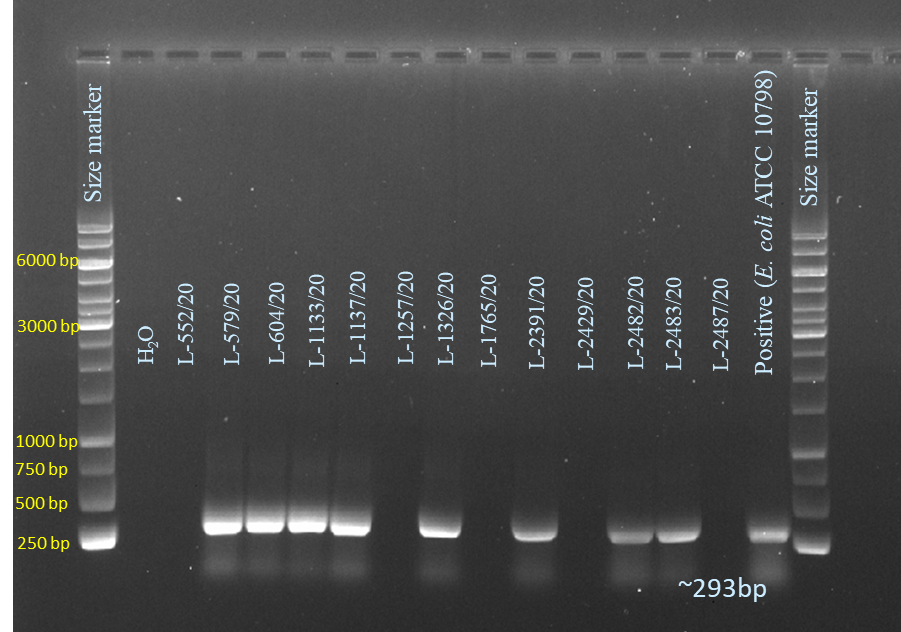
**

**Supplementary FIG 5:** Agarose (1%) gel image of PCR amplicons of 13 *Acanthamoeba* isolates targeting intracellular bacteria 16S rRNA, primer pair 515Fw and 806Rv (V4, 16S rRNA) was used which yielded ~293bp amplicons. *E. coli* (ATCC 10798) was used as a positive control for bacterial 16S rRNA. Image was captured using Gel Doc XR^+^ with Image lab Software (Bio Rad Laboratories, California, USA).
